# Supplementary material for: Characterizing the dynamics, reactivity and controllability of moods in depression with a Kalman filter
Source: PLoS Comput Biol. 2024 Sep 23;20(9):e1012457. doi: 10.1371/journal.pcbi.1012457 (PMC11449358; doi:10.1371/journal.pcbi.1012457)
Supplement: S1 Text — (PDF) [file pcbi.1012457.s001.pdf]

S1 Supplementary Material to Characterizing the dynamics, reactivity and controllability of moods in depression with a Kalman filter

Jolanda Malamud[1*], Sinan Guloksuz[2,3], Ruud van Winkel[2,4], Philippe Delespaul[2], Marc A.F. De Hert[4,5,6,7], Catherine Derom[8,9], Evert Thiery[10], Nele Jacobs[2,11], Bart P. F. Rutten[2], and Quentin J.M. Huys[1]

1 Applied Computational Psychiatry Lab, Mental Health Neuroscience Department, Division of Psychiatry and Max Planck Centre for Computational Psychiatry and Ageing Research, Queen Square Institute of Neurology, University College London, London, UK **2** Department of Psychiatry and Neuropsychology, School for Mental Health and Neuroscience, Maastricht University Medical Centre, Maastricht, The Netherlands **3** Department of Psychiatry, Yale School of Medicine, New Haven, Connecticut, USA **4** Department of Neurosciences, Centre for Clinical Psychiatry, KU Leuven, Leuven, Belgium **5** Department of Psychotic Disorders, University Psychiatric Centre KU Leuven, Kortenberg, Belgium **6** Leuven Brain Institute, KU Leuven, Leuven, Belgium **7** Antwerp Health Law and Ethics Chair, University of Antwerp, Antwerp, Belgium **8** Centre of Human Genetics, University Hospitals Leuven, KU Leuven, Leuven, Belgium **9** Department of Obstetrics and Gynecology, Ghent University Hospitals, Ghent University, Ghent, Belgium **10** Department of Neurology, Ghent University Hospital, Ghent University, Ghent, Belgium **11** Faculty of Psychology, Open University of the Netherlands, Heerlen, The Netherlands

*j.malamud@ucl.ac.uk

1 Dynamical Modelling

1.1 Kalman Filter Derivations

observation equation: $\mathbf{x}_t = \mathbf{z}_t + \boldsymbol{\eta}_t \quad \boldsymbol{\eta}_t \sim \mathcal{N}(0, \boldsymbol{\Gamma})$
dynamics equation: $\mathbf{z}_t = \mathbf{A}\mathbf{z}_{t-1} + \mathbf{h} + \mathbf{C}\mathbf{u}_t + \boldsymbol{\epsilon}_t \quad \boldsymbol{\epsilon}_t \sim \mathcal{N}(0, \boldsymbol{\Sigma})$

Noise vectors are assumed to be uncorrelated with the state and observation:
$E[\mathbf{z}_t, \boldsymbol{\epsilon}_k], E[\mathbf{z}_t, \boldsymbol{\eta}_k], E[\mathbf{x}_t, \boldsymbol{\epsilon}_k], E[\mathbf{x}_t, \boldsymbol{\eta}_k]$, and $E[\boldsymbol{\epsilon}_t, \boldsymbol{\eta}_k]$ are equal to 0.
$E[\boldsymbol{\epsilon}_t, \boldsymbol{\epsilon}_k] = 0$, if $t = k \rightarrow E[\boldsymbol{\epsilon}_k, \boldsymbol{\epsilon}_k] = \boldsymbol{\Gamma}$
$E[\boldsymbol{\eta}_t, \boldsymbol{\eta}_k] = 0$, if $t = k \rightarrow E[\boldsymbol{\eta}_k, \boldsymbol{\eta}_k] = \boldsymbol{\Sigma}$

1.1.1 Kalman Filter ($\tau \leq t$)

$$p(\mathbf{z}_t \mid \mathbf{x}^t) = \frac{\overbrace{p(\mathbf{x}_t \mid z_t)}^{N(\mathbf{z}_t, \boldsymbol{\Gamma})} p(\mathbf{z}_t \mid \mathbf{x}^{t-1})}{p(\mathbf{x}_t \mid \mathbf{x}^{t-1})} \quad \text{where} \quad p(\mathbf{z}_t \mid \mathbf{x}^{t-1}) = \int \overbrace{p(\mathbf{z}_t \mid \mathbf{z}_{t-1})}^{N(\mathbf{A}\mathbf{z}_{t-1} + \mathbf{h} + \mathbf{C}\mathbf{u}_t, \boldsymbol{\Sigma})} p(\mathbf{z}_{t-1} \mid \mathbf{x}^{t-1}) d\mathbf{z}_{t-1}$$

$$\hat{\mathbf{z}}_t^\tau = E[\mathbf{z}_t \mid \mathbf{x}^\tau]$$
$$\mathbf{P}_t^\tau = E[\tilde{\mathbf{z}}_t^\tau \tilde{\mathbf{z}}_t^\tau \mid \mathbf{x}^\tau]$$
$$\tilde{\mathbf{z}}_t^\tau = \mathbf{z}_t^\tau - \hat{\mathbf{z}}_t^\tau$$

Mean of $p(\mathbf{z}_t \mid \mathbf{x}^{t-1})$:

$$\begin{aligned}\hat{\mathbf{z}}_t^{t-1} &= E[\mathbf{z}_t \mid \mathbf{x}^{t-1}] = E[\mathbf{A}\mathbf{z}_{t-1} + \mathbf{h} + \mathbf{C}\mathbf{u}_t + \boldsymbol{\epsilon}_{t-1} \mid \mathbf{x}^{t-1}] \\ &= \mathbf{A}E[\mathbf{z}_{t-1} \mid \mathbf{x}^{t-1}] + \mathbf{h} + \mathbf{C}\mathbf{u}_t + E[\epsilon_{t-1} \mid \mathbf{x}^{t-1}] = \mathbf{A}\hat{\mathbf{z}}_{t-1}^{t-1} + \mathbf{h} + \mathbf{C}\mathbf{u}_t\end{aligned}$$

Prediction error:

$$\tilde{\mathbf{z}}_t^{t-1} = \mathbf{z}_t - \hat{\mathbf{z}}_t^{t-1} = \mathbf{A}\mathbf{z}_{t-1} + \mathbf{h} + \mathbf{C}\mathbf{u}_t + \boldsymbol{\epsilon}_{t-1} - \mathbf{A}\hat{\mathbf{z}}_{t-1}^{t-1} - \mathbf{h} - \mathbf{C}\mathbf{u}_t = \mathbf{A}\tilde{\mathbf{z}}_{t-1}^{t-1} + \boldsymbol{\epsilon}_{t-1}$$

Covariance of $p(\mathbf{z}_t \mid \mathbf{x}^{t-1})$:

$$\begin{aligned}\mathbf{P}_t^{t-1} &= E[\tilde{\mathbf{z}}_t^{t-1}\tilde{\mathbf{z}}_t^{t-1} \mid \mathbf{x}^{t-1}] = E[(\mathbf{A}\tilde{\mathbf{z}}_{t-1}^{t-1} + \boldsymbol{\epsilon}_{t-1})(\mathbf{A}\tilde{\mathbf{z}}_{t-1}^{t-1} + \boldsymbol{\epsilon}_{t-1})^T \mid \mathbf{x}^{t-1}] \\ &= E[\mathbf{A}\tilde{\mathbf{z}}_{t-1}^{t-1}(\tilde{\mathbf{z}}_{t-1}^{t-1})^T\mathbf{A}^T + 2\mathbf{A}\tilde{\mathbf{z}}_{t-1}^{t-1}\boldsymbol{\epsilon}_{t-1}^T + \boldsymbol{\epsilon}_{t-1}\boldsymbol{\epsilon}_{t-1}^T \mid \mathbf{x}^{t-1}] \\ &= \mathbf{A}E[\tilde{\mathbf{z}}_{t-1}^{t-1}(\tilde{\mathbf{z}}_{t-1}^{t-1})^T \mid \mathbf{x}^{t-1}]\mathbf{A}^T + 2\mathbf{A}E[\tilde{\mathbf{z}}_{t-1}^{t-1}\boldsymbol{\epsilon}_{t-1}^T \mid \mathbf{x}^{t-1}] + E[\boldsymbol{\epsilon}_{t-1}\boldsymbol{\epsilon}_{t-1}^T \mid \mathbf{x}^{t-1}] = \mathbf{A}\mathbf{P}_{t-1}^{t-1}\mathbf{A}^T + \boldsymbol{\Sigma}\end{aligned}$$

$$\begin{aligned}p(\mathbf{z}_t \mid \mathbf{x}^t) &= \frac{N(\hat{\mathbf{z}}_t^{t-1}, \mathbf{P}_t^{t-1})N(\mathbf{z}_t, \boldsymbol{\Gamma})}{p(\mathbf{x}_t \mid \mathbf{x}^{t-1})} \\ &\propto N(([\mathbf{P}_t^{t-1}]^{-1} + \boldsymbol{\Gamma}^{-1})^{-1}([\mathbf{P}_t^{t-1}]^{-1}\hat{\mathbf{z}}_t^{t-1} + \boldsymbol{\Gamma}^{-1}\mathbf{x}_t), ([\mathbf{P}_t^{t-1}]^{-1} + \boldsymbol{\Gamma}^{-1})^{-1})\end{aligned}$$

$$\boxed{\begin{aligned}&\hat{\mathbf{z}}_t^{t-1} = \mathbf{A}\hat{\mathbf{z}}_{t-1}^{t-1} + \mathbf{h} + \mathbf{C}\mathbf{u}_t \\ &\mathbf{P}_t^{t-1} = \mathbf{A}\mathbf{P}_{t-1}^{t-1}\mathbf{A}^T + \boldsymbol{\Sigma} \\ &\hat{\mathbf{z}}_t^t = \hat{\mathbf{z}}_t^{t-1} + \mathbf{K}_t(\mathbf{x}_t - \hat{\mathbf{z}}_t^{t-1}) \\ &\mathbf{P}_t^t = (\mathbf{I} - \mathbf{K}_t)\mathbf{P}_t^{t-1} \\ &\mathbf{K}_t = \mathbf{P}_t^{t-1}(\boldsymbol{\Gamma} + \mathbf{P}_t^{t-1})^{-1}\end{aligned}}$$

1.1.2 Kalman Smoother ($\tau > t$)

$\hat{\mathbf{z}}_{t-1}^{\tau} = E[\mathbf{z}_{t-1} \mid \mathbf{x}^{\tau}] = E[\mathbf{z}_{t-1} \mid \mathbf{z}_t = \hat{\mathbf{z}}_t^{\tau}, \mathbf{x}^{\tau}]$

$$p(\mathbf{z}_{t-1}, \mathbf{z}_t \mid \mathbf{x}^{\tau}) = \frac{p(\mathbf{z}_{t-1}, \mathbf{z}_t, \mathbf{x}^{t-1}, \mathbf{x}_t, ..., \mathbf{x}_{\tau})}{p(\mathbf{x}^{\tau})}$$
$$\propto p(\mathbf{z}_t \mid \mathbf{z}_{t-1})p(\mathbf{z}_{t-1} \mid \mathbf{x}^{t-1}) = N(\mathbf{A}\mathbf{z}_{t-1} + \mathbf{h} + \mathbf{C}\mathbf{u}_t, \mathbf{\Sigma})N(\hat{\mathbf{z}}_{t-1}^{t-1}, \mathbf{P}_{t-1}^{t-1})$$

$$p(\mathbf{z}_{t-1} \mid \mathbf{z}_t, \mathbf{x}^{\tau}) = \frac{p(\mathbf{z}_{t-1}, \mathbf{z}_t \mid \mathbf{x}^{\tau})}{p(\mathbf{z}_t \mid \mathbf{x}^{\tau})}$$
$$\propto N_{\mathbf{z}_{t-1}}((([\mathbf{P}_{t-1}^{t-1}]^{-1} + \mathbf{A}^T\mathbf{\Sigma}^{-1}\mathbf{A})^{-1}([\mathbf{P}_{t-1}^{t-1}]^{-1}\hat{\mathbf{z}}_{t-1}^{t-1} + \mathbf{A}^T\mathbf{\Sigma}^{-1}\mathbf{z}_t), ([\mathbf{P}_{t-1}^{t-1}]^{-1} + \mathbf{A}^T\mathbf{\Sigma}^{-1}\mathbf{A})^{-1})$$

$$\boxed{\begin{aligned} &\hat{\mathbf{z}}_{t-1}^{\tau} = \hat{\mathbf{z}}_{t-1}^{t-1} + \mathbf{J}_{t-1}(\hat{\mathbf{z}}_t^{\tau} - \hat{\mathbf{z}}_t^{t-1}) \\ &\mathbf{J}_{t-1} = \mathbf{P}_{t-1}^{t-1}\mathbf{A}^T[\mathbf{P}_t^{t-1}]^{-1} \\ &\mathbf{P}_{t-1}^{\tau} = \mathbf{P}_{t-1}^{t-1} + \mathbf{J}_{t-1}(\mathbf{P}_t^{\tau} - \mathbf{P}_t^{t-1})\mathbf{J}_{t-1}^T \end{aligned}}$$

Lag-one covariance smoother:

$\mathbf{P}_{\tau,\tau-1}^{\tau} = (\mathbf{I} - \mathbf{K}_{\tau})\mathbf{A}\mathbf{P}_{\tau-1}^{\tau-1}$
$\mathbf{P}_{t-1,t-2}^{\tau} = \mathbf{P}_{t-1}^{t-1}(vJ_{t-2})^T + \mathbf{J}_{t-1}(\mathbf{P}_{t,t-1}^{\tau} - \mathbf{A}\mathbf{P}_{t-1}^{t-1})(\mathbf{J}_{t-2})^T$

$$\boxed{\begin{aligned} &E[\mathbf{z}_t \mid \mathbf{x}^{\tau}] = \hat{\mathbf{z}}_t^{\tau} \\ &E[\mathbf{z}_t\mathbf{z}_t^T \mid \mathbf{x}^{\tau}] = \mathbf{P}_t^{\tau} + \hat{\mathbf{z}}_t^{\tau}\hat{\mathbf{z}}_t^{\tau} \\ &E[\mathbf{z}_t\mathbf{z}_{t-1}^T \mid \mathbf{x}^{\tau}] = \mathbf{P}_{t,t-1}^{\tau} + \hat{\mathbf{z}}_t^{\tau}\hat{\mathbf{z}}_{t-1}^{\tau} \end{aligned}}$$

1.2 EM-Algorithm

$$L(\boldsymbol{\theta}) = logP(\mathbf{X} \mid \boldsymbol{\theta}) = log\int_{\mathbf{Z}} P(\mathbf{X}, \mathbf{Z} \mid \theta)d\mathbf{Z}$$
$$log\int_{\mathbf{Z}} Q(\mathbf{Z})\frac{P(\mathbf{X}, \mathbf{Z} \mid \boldsymbol{\theta})}{Q(\mathbf{Z})}d\mathbf{Z} \leq \int_{\mathbf{Z}} Q(\mathbf{Z})log\frac{P(\mathbf{X}, \mathbf{Z} \mid \boldsymbol{\theta})}{Q(\mathbf{Z})}d\mathbf{Z}$$
$$= \int_{\mathbf{Z}} Q(\mathbf{Z})logP(\mathbf{X}, \mathbf{Z} \mid \boldsymbol{\theta})d\mathbf{X} - \int_{\mathbf{Z}} Q(\mathbf{Z})logQ(\mathbf{Z})d\mathbf{Z} = F(Q, \boldsymbol{\theta})$$

E-step: $Q_{k+1} \leftarrow \underset{Q}{argmax}F(Q, \boldsymbol{\theta}_k)$ Maximum in the E-step results when Q is exactly the conditional distribution of $\mathbf{X}$: $Q_{k+1}(\mathbf{Z}) = P(\mathbf{Z} \mid \mathbf{X}, \boldsymbol{\theta}_k)$, at which point the bound becomes an equality: $F(Q_{k+1}, \boldsymbol{\theta}_k) = L(\boldsymbol{\theta}_k)$

M-step: $\boldsymbol{\theta}_{k+1} \leftarrow \underset{\boldsymbol{\theta}}{argmax}F(Q_{k+1}, \boldsymbol{\theta})$ The maximum in the M-step is obtained by maximizing the first term since the entropy of Q does not depend on $\boldsymbol{\theta}$:
$\boldsymbol{\theta}_{k+1} \leftarrow \underset{\boldsymbol{\theta}}{argmax} \int_{\mathbf{Z}} P(\mathbf{Z} \mid \mathbf{X}, \boldsymbol{\theta}_k)logP(\mathbf{Z}, \mathbf{X} \mid \boldsymbol{\theta}_k)d\mathbf{Z}$

1.2.1 Likelihoods

Joint loglikelihood $p(\mathbf{Z}, \mathbf{X} \mid \boldsymbol{\theta})$:

$$
\begin{aligned}
logp(\mathbf{z}^\tau, \mathbf{x}^\tau) =& log[\underbrace{p(\mathbf{z}_1)}_{N(\boldsymbol{\mu}_0,\boldsymbol{\Sigma})}]\sum_{t=2}^{\tau} log[\underbrace{p(\mathbf{z}_t \mid \mathbf{z}_{t-1})}_{N(\mathbf{A}\mathbf{z}_{t-1}+\mathbf{h}+\mathbf{C}\mathbf{u}_t,\boldsymbol{\Sigma})}]\sum_{t=1}^{\tau} log[\underbrace{p(\mathbf{x}_t \mid \mathbf{z}_t)}_{N(\mathbf{z}_t,\boldsymbol{\Gamma})}] \\
=& -\frac{1}{2}[log(\mid 2\pi\boldsymbol{\Sigma} \mid) + (\mathbf{z}_1 - \boldsymbol{\mu}_0 - \mathbf{C}\mathbf{u}_1)^T\boldsymbol{\Sigma}^{-1}(\mathbf{z}_1 - \boldsymbol{\mu}_0 - \mathbf{C}\mathbf{u}_1) \\
&+ (\tau - 1)log(\mid 2\pi\boldsymbol{\Sigma} \mid) + \sum_{t=2}^{\tau}(\mathbf{z}_t - \mathbf{A}\mathbf{z}_{t-1} - \mathbf{h} - \mathbf{C}\mathbf{u}_t)^T\boldsymbol{\Sigma}^{-1}(\mathbf{z}_t - \mathbf{A}\mathbf{z}_{t-1} - \mathbf{h} - \mathbf{C}\mathbf{u}_t) \\
&+ \tau log(\mid 2\pi\boldsymbol{\Gamma} \mid) + \sum_{t=2}^{\tau}(\mathbf{x}_t - \mathbf{z}_t)^T\boldsymbol{\Gamma}^{-1}(\mathbf{x}_t - \mathbf{z}_t)]
\end{aligned}
$$

Marginal loglikelihood $p(\mathbf{X} \mid \boldsymbol{\theta})$:

$$logp(\mathbf{x}^\tau) = \sum_{t=1}^{\tau} logp(\mathbf{x}_t \mid \mathbf{x}^{t-1}) + logp(\mathbf{x}_1)$$

$$
\begin{aligned}
E[p(\mathbf{x}_t \mid \mathbf{x}^{t-1})] &= \int p(\mathbf{x}_t \mid \mathbf{x}^{t-1})\mathbf{x}_t d\mathbf{x}_t = \int\int \underbrace{p(\mathbf{x}_t \mid \mathbf{z}_t)}_{N_{\mathbf{x}_t}(\mathbf{z}_t,\boldsymbol{\Gamma})} p(\mathbf{z}_t \mid \mathbf{x}^{t-1})\mathbf{x}_t d\mathbf{z}_t d\mathbf{x}_t = \int p(\mathbf{z}_t \mid \mathbf{x}^{t-1})\mathbf{z}_t d\mathbf{z}_t \\
&= E[\mathbf{z}_t \mid \mathbf{x}^{t-1}] = \hat{\mathbf{z}}_t^{t-1} = \mathbf{A}\hat{\mathbf{z}}_{t-1}^{t-1}
\end{aligned}
$$

$$E[p(\mathbf{x}_1)] = \boldsymbol{\mu}_0$$

$$
\begin{aligned}
Cov[p(\mathbf{x}_t \mid \mathbf{x}^{t-1})] &= \int p(\mathbf{x}_t \mid \mathbf{x}^{t-1})(\mathbf{x}_t\mathbf{x}_t^T - E[\mathbf{x}_t \mid \mathbf{x}^{t-1}]E[\mathbf{x}_t \mid \mathbf{x}^{t-1}]^T)d\mathbf{x}_t \\
&= \int\int \underbrace{p(\mathbf{x}_t \mid \mathbf{z}_t)}_{N_{\mathbf{x}_t}(\mathbf{z}_t,\boldsymbol{\Gamma})} p(\mathbf{z}_t \mid \mathbf{x}^{t-1})(\mathbf{x}_t\mathbf{x}_t^T - E[\mathbf{x}_t \mid \mathbf{x}^{t-1}]E[\mathbf{x}_t \mid \mathbf{x}^{t-1}]^T)d\mathbf{z}_t d\mathbf{x}_t \\
&= \int p(\mathbf{z}_t \mid \mathbf{x}^{t-1})(\boldsymbol{\Gamma} + \mathbf{z}_t\mathbf{z}_t^T - E[\mathbf{x}_t \mid \mathbf{x}^{t-1}]E[\mathbf{x}_t \mid \mathbf{x}^{t-1}]^T)d\mathbf{z}_t \\
&= \int p(\mathbf{z}_t \mid \mathbf{x}^{t-1})(\boldsymbol{\Gamma} + \mathbf{z}_t\mathbf{z}_t^T - \hat{\mathbf{z}}_t^{t-1}\hat{\mathbf{z}}_t^{t-1})d\mathbf{z}_t \\
&= \boldsymbol{\Gamma} + (\mathbf{P}_t^{t-1} + \hat{\mathbf{z}}_t^{t-1}\hat{\mathbf{z}}_t^{t-1}) - \hat{\mathbf{z}}_t^{t-1}\hat{\mathbf{z}}_t^{t-1} = \boldsymbol{\Gamma} + \mathbf{P}_t^{t-1}
\end{aligned}
$$

$$Cov[p(x_1)] = \boldsymbol{\Gamma} + \boldsymbol{\Sigma}$$

$$logp(\mathbf{X} \mid \boldsymbol{\theta}) = -\frac{1}{2}[\tau log(2\pi\sum_{t=1}^{\tau}(\boldsymbol{\Gamma} + \mathbf{P}_t^{t-1})) + \sum_{t=1}^{\tau}(\mathbf{x}_t - \mathbf{A}\hat{\mathbf{z}}_{t-1}^{t-1})^T(\boldsymbol{\Gamma} + \mathbf{P}_t^{t-1})^{-1}(\mathbf{x}_t - \mathbf{A}\hat{\mathbf{z}}_{t-1}^{t-1})]$$

1.2.2 Parameter estimation

Solve $\mathbf{A}$, $\mathbf{h}$ and $\mathbf{C}$ in one go:

$\mathbf{L} = [\mathbf{A}, \mathbf{h}, \mathbf{C}]$
$\mathbf{o}_t = [\mathbf{z}_{t-1}; \mathbf{1}; \mathbf{u_t}]$
$\mathbf{L}\mathbf{o}_t = \mathbf{A}\mathbf{z}_{t-1} + \mathbf{h} + \mathbf{C}\mathbf{u}_t$

$$\begin{aligned}\frac{\partial log[P(\mathbf{Z},\mathbf{X}\mid\boldsymbol{\theta})]}{\partial\mathbf{L}} &= \frac{\partial}{\partial\mathbf{L}}(-\frac{1}{2}\sum_{t=2}^{\tau}(\mathbf{z}_t-\mathbf{L}\mathbf{o}_t)^T\mathbf{\Sigma}^{-1}(\mathbf{z}_t-\mathbf{L}\mathbf{o}_t))\\ &= \frac{\partial}{\partial\mathbf{L}}(-\frac{1}{2}\sum_{t=2}^{\tau}(\mathbf{o}_t^T\mathbf{L}^T\mathbf{\Sigma}^{-1}\mathbf{L}\mathbf{o}_t-2\mathbf{o}_t^T\mathbf{L}^T\mathbf{\Sigma}^{-1}\mathbf{z}_t))\\ &= -\frac{1}{2}\sum_{t=2}^{\tau}(2\mathbf{\Sigma}^{-1}\mathbf{o}_t\mathbf{o}_t^T-2\mathbf{\Sigma}^{-1}\mathbf{z}_t\mathbf{o}_t^T)\end{aligned}$$

$$\rightarrow\mathbf{L}=(\sum_{t=2}^{\tau}E[\mathbf{z}_t\mathbf{o}_t^T\mid\mathbf{x}^\tau])(\sum_{t=2}^{\tau}E[\mathbf{o}_t\mathbf{o}_t^T\mid\mathbf{x}^\tau])^{-1}$$

$$\sum_{t=2}^{\tau}E[\mathbf{z}_t\mathbf{o}_t^T\mid\mathbf{x}^\tau]=\begin{bmatrix}\sum_{t=2}^{\tau}E[\mathbf{z}_t\mathbf{z}_{t-1}^T\mid\mathbf{x}^\tau] & \sum_{t=2}^{\tau}E[\mathbf{z}_t\mid\mathbf{x}^\tau] & \sum_{t=2}^{\tau}E[\mathbf{z}_t\mid\mathbf{x}^\tau]\mathbf{u}_t^T\end{bmatrix}$$

$$\sum_{t=2}^{\tau}E[\mathbf{o}_t\mathbf{o}_t^T\mid\mathbf{x}^\tau]=\begin{bmatrix}\sum_{t=2}^{\tau}E[\mathbf{z}_{t-1}\mathbf{z}_{t-1}^T\mid x^\tau] & \sum_{t=2}^{\tau}E[\mathbf{z}_{t-1}\mid\mathbf{x}^\tau] & \sum_{t=2}^{\tau}E[\mathbf{z}_{t-1}\mid\mathbf{x}^\tau]s_t^T\\ \sum_{t=2}^{\tau}E[\mathbf{z}_{t-1}^T\mid\mathbf{x}^\tau] & (\tau-1) & \sum_{t=2}^{\tau}\mathbf{u}_t^T\\ \sum_{t=2}^{\tau}\mathbf{u}_tE[\mathbf{z}_{t-1}^T\mid\mathbf{x}^\tau] & \sum_{t=2}^{\tau}\mathbf{u}_t & \sum_{t=2}^{\tau}\mathbf{u}_t\mathbf{u}_t^T\end{bmatrix}$$

$$\begin{aligned}\mathbf{\Sigma}=&\frac{1}{\tau}[-\mathbf{z}_1(\boldsymbol{\mu}_0+\mathbf{C}\mathbf{u}_1)^T-(\boldsymbol{\mu}_0+\mathbf{C}\mathbf{u}_1)\mathbf{z}_1^T+\boldsymbol{\mu}_0\mathbf{u}_1^T\mathbf{C}^T+\mathbf{C}\mathbf{u}_1\boldsymbol{\mu}_0^T+\boldsymbol{\mu}_0\boldsymbol{\mu}_0^T\\ &+\sum_{t=1}^{\tau}(E[\mathbf{z}_t\mathbf{z}_t^T\mid\mathbf{x}^\tau]-E[\mathbf{z}_t\mid\mathbf{x}^\tau]\mathbf{u}_t^T\mathbf{C}^T-\mathbf{C}\mathbf{u}_tE[\mathbf{z}_t\mid\mathbf{x}^\tau]^T+\mathbf{C}\sum_{t=1}^{\tau}\mathbf{u}_t\mathbf{u}_t^T\mathbf{C}^T)\\ &+\sum_{t=2}^{\tau}(-\mathbf{A}E[\mathbf{z}_t\mathbf{z}_{t-1}^T\mid\mathbf{x}^\tau]^T-E[\mathbf{z}_t\mathbf{z}_{t-1}^T\mid\mathbf{x}^\tau]\mathbf{A}^T+\mathbf{A}E[\mathbf{z}_{t-1}\mathbf{z}_{t-1}^T\mid\mathbf{x}^\tau]\mathbf{A}^T\\ &+\mathbf{A}E[\mathbf{z}_{t-1}\mid\mathbf{x}^\tau]\mathbf{h}^T+\mathbf{h}E[\mathbf{z}_{t-1}\mid\mathbf{x}^\tau]^T\mathbf{A}^T+\mathbf{A}E[\mathbf{z}_{t-1}\mid\mathbf{x}^\tau]\mathbf{u}_t^T\mathbf{C}^T\\ &+\mathbf{C}\mathbf{u}_tE[\mathbf{z}_{t-1}\mid\mathbf{x}^\tau]^T\mathbf{A}^T-\mathbf{h}E[\mathbf{z}_t\mid\mathbf{x}^\tau]^T-E[\mathbf{z}_t\mid\mathbf{x}^\tau]\mathbf{h}^T+\mathbf{h}\mathbf{h}^T+\mathbf{h}\mathbf{u}^T\mathbf{C}^T+\mathbf{C}\mathbf{u}_t\mathbf{h}^T)]\end{aligned}$$

$$\mathbf{\Gamma}=\frac{1}{\tau}\sum_{t=1}^{\tau}(\mathbf{x}_t\mathbf{x}_t^T-\mathbf{x}_tE[\mathbf{z}_t\mid\mathbf{x}^\tau]^T-E[\mathbf{z}_t\mid\mathbf{x}^\tau]\mathbf{x}_t^T+E[\mathbf{z}_t\mathbf{z}_t^T\mid\mathbf{x}^\tau])$$

Lasso-Regularization for C:

$$\begin{aligned}logp(\mathbf{X}\mid\boldsymbol{\theta})=&-\frac{1}{2}[\tau log(2\pi\sum_{t=1}^{\tau}(\mathbf{\Gamma}+\mathbf{P}_t^{t-1}))+\sum_{t=1}^{\tau}(\mathbf{x}_t-\mathbf{A}\hat{\mathbf{z}}_{t-1}^{t-1})^T(\mathbf{\Gamma}+\mathbf{P}_t^{t-1})^{-1}(\mathbf{x}_t-\mathbf{A}\hat{\mathbf{z}}_{t-1}^{t-1})]\\ &-r\sum_{i=1,j=1}^{d}\mid\mathbf{C}_{ij}\mid\end{aligned}$$

1.3 Optimal Control

We exploited control theory to find the optimal inputs $\mathbf{u}^*$ to steer the mood state into a specific direction. We explored a possible application of optimal control [1] using a linear quadratic regulator. The objective of linear quadratic regulator is to minimize the cost associated with the error in the system state, while also minimizing the cost of actuator effort. Hence, $\mathbf{J}_i$ (cf. Equation 1) is minimized subject to the

constraint of the state equation to find the input $\mathbf{u}_k$ on the interval $[i, N]$ (i.e., $\mathbf{u}_k, \forall k \in [i, N]$) that drives the system along a trajectory which converges to a healthy state.

$$\mathbf{J}_i(\mathbf{z}, \mathbf{u}) = \frac{1}{2}(\mathbf{z}_i - \mathbf{r}_N)^T(\mathbf{z}_i - \mathbf{r}_N) + \sum_{k=i}^{N}[\mathbf{z}_k^T\mathbf{Q}\mathbf{z}_k + \mathbf{u}_k^T\mathbf{R}\mathbf{u}_k] \tag{1}$$

$$\mathbf{P}_{i-1} = \mathbf{A}^T(\mathbf{P}_i + \mathbf{C}\mathbf{R}^{-1}\mathbf{C}^T)^{-1}\mathbf{A} + \mathbf{Q} \tag{2}$$

$$\mathbf{K}_i = -(\mathbf{R} + \mathbf{C}^T\mathbf{P}_{i+1}\mathbf{C})^{-1}\mathbf{C}^T\mathbf{P}_{i+1}\mathbf{A} \tag{3}$$

$$\mathbf{u}_i = \mathbf{K}_i(\mathbf{z}_i - \mathbf{r}_N) \tag{4}$$

We aim to achieve the system to stabilize at the desired state $\mathbf{r}_N$. $\mathbf{R}$ is the control input cost matrix, which penalizes the amount of effort required. $\mathbf{Q}$ is the state cost matrix, weighing the importance of each state in the state vector. By assigning high values to specific elements in $\mathbf{R}$, respectively $\mathbf{Q}$, we can prioritize control inputs that require less effort, respectively states that require less correction. However, this remains a topic for future investigation since we used an identity matrix for both $\mathbf{R}$ and $\mathbf{Q}$, treating all inputs and states equally. $\mathbf{P}$ is the discrete time algebraic Riccati equation [1] and $\mathbf{K}$ holds the optimal feedback gain values.

Solutions to discrete linear quadratic regulator problems are obtained using dynamic programming. The optimal solution is computed recursively, beginning at the last timestep and moving backwards in time. Further details can be found in subsection 1.3 Optimal Control.

Dynamic programming algorithm for linear quadratic regulator:

1) set $\mathbf{P}_N = \mathbf{Q}$
2) for $i = N, ..., 1$: $\mathbf{P}_{i-1} = \mathbf{Q} + \mathbf{A}^T\mathbf{P}_i\mathbf{A} - \mathbf{A}^T\mathbf{P}_i\mathbf{C}(\mathbf{R} + \mathbf{C}^T\mathbf{P}_i\mathbf{C})^{-1}\mathbf{C}^T\mathbf{P}_i\mathbf{A}$
3) for $i = 0, ..., N-1$: $\mathbf{K}_i = -(\mathbf{R} + \mathbf{C}^T\mathbf{P}_{i+1}\mathbf{C})^{-1}\mathbf{C}^T\mathbf{P}_{i+1}\mathbf{A}$
4) for $i = 0, ..., N-1$: $\mathbf{u}_i = \mathbf{K}_i(\mathbf{z}_i - \mathbf{r}_N)$

2 Datasets

Dataset 1 [2] consisted of experience sampling data including four mood items (cheerful, content, anxious, and sad) acquired over five or six consecutive days. The dataset comprised two groups; 1) depressed patient group ($N = 150$) and 2) healthy control group ($N = 579$). Subjects received a digital wristwatch that indicated by a beep when subjects were asked to complete a paper-based experience sampling form. Subjects were required to complete this form 10 times per day at random moments within 90-min time blocks during daytime. This resulted in a maximum of 50 or 60 measurements of four different mood scores on seven-point Likert scales. We excluded participants if they had less than 5 days of experience sampling data, had missed one or more full days of experience sampling, and less than 40 measurements points in total (corresponding to a completion rate of approximately 75%). Additionally, participants who showed no variability in all mood items were excluded. In total, 402 participants ($N_{\text{patients}} = 33$, $N_{\text{controls}} = 369$) were excluded. The final sample consisted of 117 in the depressed group and 210 in the healthy group. Patients completed on average 50 ($SD = 5$) beeps (cheerfulness ratings: M [1] $= 2.87$, $SD = 1.55$; content ratings: $M = 3.06$, $SD = 1.59$; anxiety ratings: $M = 1.68$, $SD = 1.23$; sadness ratings: $M = 2.36$, $SD = 1.3$) and controls completed 43 ($SD = 3$) beeps (cheerfulness ratings: $M = 4.68$, $SD = 0.95$; content ratings: $M = 4.85$, $SD = 0.93$; anxiety ratings: $M = 1.18$, $SD = 0.29$; sadness ratings: $M = 1.32$, $SD = 0.46$).

Dataset 2 came from the TwinssCan project (the detailed procedure for the TwinssCan study is fully described elsewhere [3]). The data consisted of 839 participants. The experience sampling measures were

[1] M = average of mean ratings over sample; SD = standard deviation of mean ratings

administered using Psy-mate, a custom-made Personal Digital Assistant [4], which emitted a notification prompting participants to complete the questionnaire. The surveys included questions about participants mood probing various items, what they were doing, where they were and whom they were with at that moment. Notifications were given between 7:30am and 10:30pm. During a baseline and one-year follow-up, participants completed the self-report Symptom Checklist 90 (SCL-90-R [5]) including questions regarding experiences of psychopathology symptoms. We chose the same mood items (cheerful, content, anxious, and sad) as in dataset 1 to be able to draw conclusion across datasets. After excluding participants ($N = 400$) due to the same exclusion criteria as in dataset 1, the final sample included 439 participants. Participants completed on average 49 ($SD = 6$) beeps (cheerfulness ratings: $M = 4.83$, $SD = 0.85$; content ratings: $M = 5.14$, $SD = 0.66$; anxiety ratings: $M = 1.72$, $SD = 0.64$; sadness ratings: $M = 1.48$, $SD = 0.5$). SCL total score at baseline was on average 0.56 ($SD = 0.53$) and at follow-up 0.52 ($SD = 0.5$).

Dataset 3 [6] consisted of only one participant, of which momentary observations of daily life experiences were collected at 1,472 time points. The participant was diagnosed with major depression disorder and had been using antidepressants for several years (for details, see [7]). Using a semi-random experience-sampling protocol, the participant was asked to report his momentary experiential states 10 times a day over a period of 239 days, including gradual discontinuation of his antidepressant medication. In addition to mood, questions combined with different options to choose from about what the participant was doing at that moment, and who he was with, were part of the experience sampling procedure. We again chose the time-series of those four items (cheerful, content, anxious, and sad) and separated them into three parts: 1) 6 weeks of experience sampling before the tapering of the antidepressant started as a baseline measure (number of completed beeps= 176; cheerfulness ratings: m [2] $= 4.06$, $std = 0.76$; content ratings: $m = 4.11$, $std = 0.93$; anxiety ratings: $m = 3.9$, $std = 0.34$; sadness ratings: $m = 4.07$, $std = 0.57$; SCL total score= 1.26) 2) 8 weeks during discontinuation (number of completed beeps= 176; cheerfulness ratings: $m = 4.11$, $std = 0.83$; content ratings: $m = 4.29$, $std = 1$; anxiety ratings: $m = 3.87$, $std = 0.35$; sadness ratings: $m = 4.04$, $std = 0.64$; SCL total score= 1.23); 3) 6 weeks after the transition occurred (number of completed beeps= 176; cheerfulness ratings: $m = 4.07$, $std = 0.89$; content ratings: $m = 4.15$, $std = 1.01$; anxiety ratings: $m = 3.95$, $std = 0.23$; sadness ratings: $m = 4.37$, $std = 0.81$; SCL total score= 1.93). According to the weekly assessed SCL-90 measurements, a sudden shift in depressive symptoms was observed around day 127 of the experiment. [8] conducted a change point analysis, which confirmed a statistically significant transition at that point. They found that temporal autocorrelation and variance of mood items increased before the transition [2].

[2] m = mean rating averaged over individual time-series; std = standard deviation of individual time-series

3 Additional Figures & Tables

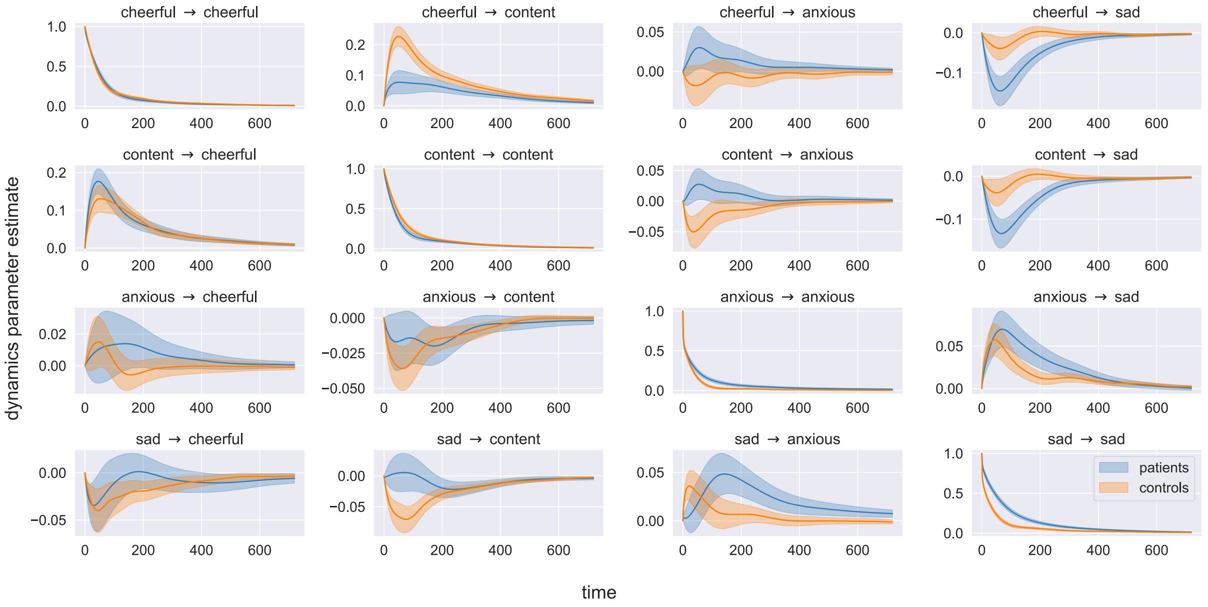

Figure A: Estimated dynamics matrix elements evolving over time (in minutes; A^{time}) averaged over participants (line) ± standard error (shaded). Orange are controls and blue are patients.

	patients (M ± SD)	controls (M ± SD)	U	pvalue
cheerful → cheerful (a_{11})	0.35 ± 0.36	0.32 ± 0.46	12607	0.69
content → cheerful (a_{12})	0.08 ± 0.41	0.22 ± 0.47	9659	0.001
anxious → cheerful (a_{13})	0.03 ± 0.29	-0.02 ± 0.36	13173	0.28
sad → cheerful (a_{14})	-0.15 ± 0.4	-0.04 ± 0.42	10293	0.01
cheerful → content (a_{21})	0.17 ± 0.36	0.13 ± 0.52	13345	0.20
content → content (a_{22})	0.3 ± 0.38	0.38 ± 0.45	10276	0.01
anxious → content (a_{23})	0.03 ± 0.27	-0.04 ± 0.38	13494	0.14
sad → content (a_{24})	-0.13 ± 0.37	-0.04 ± 0.45	9536	<0.001
cheerful → anxious (a_{31})	0.01 ± 0.24	0.01 ± 0.21	12269	0.98
content → anxious (a_{32})	-0.02 ± 0.22	-0.04 ± 0.22	12429	0.86
anxious → anxious (a_{33})	0.21 ± 0.29	0.12 ± 0.26	14367	0.01
sad → anxious (a_{34})	0.07 ± 0.24	0.05 ± 0.27	12574	0.72
cheerful → sad (a_{41})	-0.03 ± 0.34	-0.04 ± 0.32	12056	0.78
content → sad (a_{42})	0.01 ± 0.34	-0.07 ± 0.33	14065	0.03
anxious → sad (a_{43})	0.02 ± 0.27	0.03 ± 0.29	12457	0.83
sad → sad (a_{44})	0.42 ± 0.36	0.19 ± 0.35	16797	<0.001

Table A: Comparing dynamics matrix elements between patients and controls. The original estimates from the state space model, in which discrete time evolves in minutes, were transformed to an hourly estimate (A^{60}). We report here the mean (M) and standard deviation (SD) of the parameter point estimates and the results of Mann-Whitney U tests comparing patients and healthy controls.

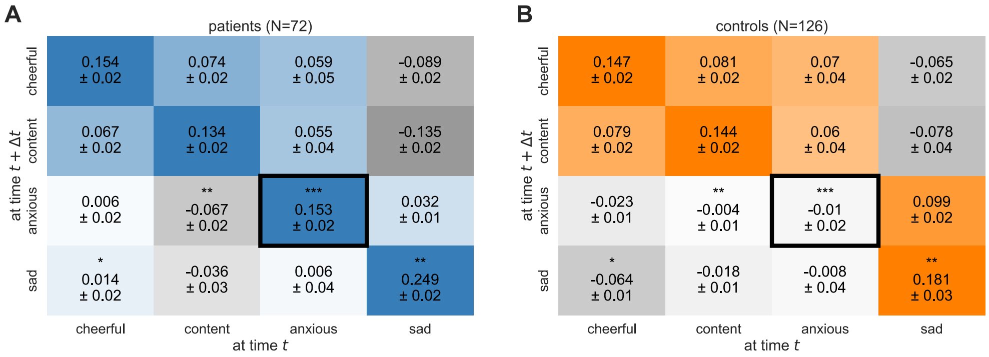

FIGURE B: **Differences in vector autoregressive (VAR) matrices between Patients and Controls**. **A)** shows the estimates of the VAR elements averaged over patients (N=72; M ± SEM). **B)** shows the estimates of the VAR matrix averaged over healthy controls (N=126; M ± SEM). The black frame indicates the significant difference between estimates after correcting for multiple comparison. The observed patterns here are less clear compared to the matrices estimated through the Kalman Filter, with the mutual enhancement of positive feelings (cheerfulness and contentedness) and the reduction of negative feelings (anxiety and sadness) being less evident.

	patients (M ± SD)	controls (M ± SD)	U	pvalue
cheerful → cheerful (a_{11})	0.15 ± 0.23	0.15 ± 0.24	4579	0.91
content → cheerful (a_{12})	0.07 ± 0.23	0.08 ± 0.23	4516	0.96
anxious → cheerful (a_{13})	0.06 ± 0.57	0.07 ± 0.53	4054	0.21
sad → cheerful (a_{14})	-0.09 ± 0.22	-0.06 ± 0.36	4456	0.84
cheerful → content (a_{21})	0.07 ± 0.23	0.08 ± 0.26	4416	0.76
content → content (a_{22})	0.13 ± 0.23	0.14 ± 0.25	4407	0.74
anxious → content (a_{23})	0.05 ± 0.43	0.06 ± 0.65	4397	0.72
sad → content (a_{24})	-0.13 ± 0.27	-0.08 ± 0.54	3914	0.11
cheerful → anxious (a_{31})	0.01 ± 0.22	-0.02 ± 0.15	4986	0.25
content → anxious (a_{32})	-0.07 ± 0.25	-0.0 ± 0.16	3458	0.005
anxious → anxious (a_{33})	0.15 ± 0.25	-0.01 ± 0.34	5933	<0.001
sad → anxious (a_{34})	0.03 ± 0.15	0.1 ± 0.34	4335	0.60
cheerful → sad (a_{41})	0.01 ± 0.26	-0.06 ± 0.17	5378	0.03
content → sad (a_{42})	-0.04 ± 0.31	-0.02 ± 0.18	4241	0.45
anxious → sad (a_{43})	0.01 ± 0.41	-0.01 ± 0.56	4947	0.29
sad → sad (a_{44})	0.25 ± 0.24	0.18 ± 0.39	5538	0.01

TABLE B: Comparing vector autoregressive (VAR) matrix elements between patients and controls. We report here the mean (M) and standard deviation (SD) of the parameter point estimates and the results of Mann-Whitney U tests comparing patients and healthy controls.

		patients (M ± SD)	controls (M ± SD)	*U*	pvalue
eigenvalues	1st	0.99 ± 0.01	0.99 ± 0.01	13886.0	0.051
	2nd	0.97 ± 0.08	0.98 ± 0.02	13298.0	0.217
	3rd	0.87 ± 0.25	0.85 ± 0.27	14261.0	0.016
	4th	0.68 ± 0.33	0.66 ± 0.33	13520.0	0.132
	overall stability	0.63 ± 0.36	0.59 ± 0.37	13524.0	0.131
slowest eigenvector	cheerful	0.39 ± 0.35	0.54 ± 0.3	9215.0	<0.001
	content	0.4 ± 0.32	0.53 ± 0.28	9599.0	0.001
	anxious	-0.16 ± 0.32	-0.09 ± 0.24	11654.0	0.442
	sad	-0.36 ± 0.39	-0.22 ± 0.31	9738.0	0.002
variability	cheerful	0.86 ± 0.66	1.16 ± 0.82	9443.0	<0.001
	content	0.86 ± 0.72	1.37 ± 0.94	7740.0	<0.001
	anxious	0.57 ± 0.98	0.4 ± 0.61	12343.0	0.943
	sad	0.94 ± 0.9	0.61 ± 0.81	15600.0	<0.001
instability	cheerful	1.0 ± 0.44	1.22 ± 0.4	9000.5	<0.001
	content	1.0 ± 0.4	1.37 ± 0.5	7177.5	<0.001
	anxious	0.59 ± 0.59	0.59 ± 0.57	11964.5	0.692
	sad	0.95 ± 0.57	0.69 ± 0.61	15425.5	<0.001
inertia	cheerful	0.25 ± 0.21	0.23 ± 0.18	4820.0	0.526
	content	0.24 ± 0.21	0.21 ± 0.18	4841.0	0.492
	anxious	0.2 ± 0.23	0.07 ± 0.2	6101.5	<0.001
	sad	0.29 ± 0.21	0.22 ± 0.25	5444.0	0.026
mean	cheerful	2.87 ± 1.55	4.68 ± 0.95	4531.0	<0.001
	content	3.06 ± 1.59	4.85 ± 0.93	4733.0	<0.001
	anxious	1.68 ± 1.22	1.18 ± 0.29	13226.5	0.243
	sad	2.36 ± 1.38	1.32 ± 0.46	18080.5	<0.001

TABLE C: Comparing dynamical features between patients and controls. We report mean (M) and standard deviation (SD) of the dynamical features and the results of Mann-Whitney U test comparing patients and healthy controls. Eigenvalues and eigenvectors are derived from the estimated dynamics matrix of the state space model for each participant. Instability refers to frequent and abrupt variability in an individual's time-series, using root mean squared successive difference (RMSSD), inertia refers to the autoregressive coefficient of an individual's time-series, and the mean was averaged over an individual's time-series.

		r	pvalue
eigenvalues	1st	0.13	0.007
	2nd	0.15	0.002
	3rd	0.25	<0.001
	4th	0.29	<0.001
	overall stability	0.29	<0.001
slowest eigenvector	cheerful	-0.14	0.005
	content	-0.07	0.164
	anxious	0.19	<0.001
	sad	0.13	0.006
fastest eigenvector	cheerful	0.14	0.003
	content	0.16	<0.001
	anxious	0.03	0.548
	sad	-0.14	0.003
singular values control	cheerful	-0.03	0.487
	content	-0.00	0.982
	anxious	0.04	0.411
	sad	0.12	0.017
most controllable direction	cheerful	-0.13	0.008
	content	-0.15	0.002
	anxious	0.30	<0.001
	sad	0.13	0.006
variability	cheerful	0.08	0.106
	content	0.08	0.094
	anxious	0.37	<0.001
	sad	0.36	<0.001
instability	cheerful	0.07	0.129
	content	0.04	0.357
	anxious	0.34	<0.001
	sad	0.34	<0.001
inertia	cheerful	0.02	0.669
	content	0.08	0.089
	anxious	0.21	<0.001
	sad	0.15	0.003
mean	cheerful	-0.31	<0.001
	content	-0.40	<0.001
	anxious	0.45	<0.001
	sad	0.39	<0.001

TABLE D: Comparing links between dynamical features and depression score using spearman correlation. We report correlation coefficient (r) and the corresponding pvalues. Eigenvalues and eigenvectors are derived from the estimated dynamics matrix of the state space model for each participant. Instability refers to frequent and abrupt variability in an individual's time-series, using root mean squared successive difference (RMSSD), inertia refers to the autoregressive coefficient of an individual's time-series, and the mean was averaged over an individual's time-series.

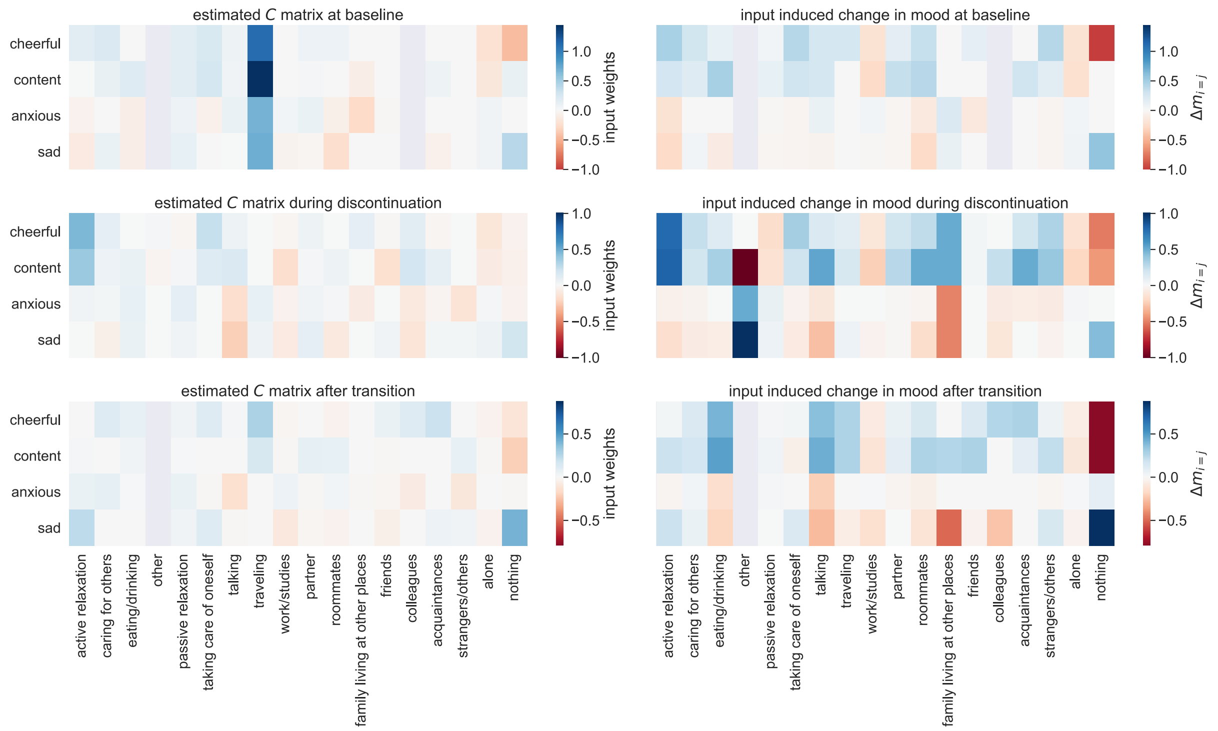

FIGURE C: To the left, heatmaps of the estimated input weights (C) are shown for the three different phases (at baseline, during discontinuation and after transitioning to a more severely depressed state). To the right, heatmaps of average changes in mood ratings for specific inputs are shown. Those changes were inferred by regressing ratings at a time-point where a specific input took place to the ratings at the previous time-point. Note that we used a lasso regularization for the estimation with a penalty factor of 2 to the input weights $\mathbf{C}$. The penalty value was set by hand.

REFERENCES

[1] Lewis FL, Vrabie DL, Syrmos VL. Optimal Control of Discrete-Time Systems. In: Optimal Control. John Wiley & Sons, Ltd; 2012. p. 19-109.

[2] Leemput IAvd, Wichers M, Cramer AOJ, Borsboom D, Tuerlinckx F, Kuppens P, et al. Critical slowing down as early warning for the onset and termination of depression. Proc Natl Acad Sci USA. 2014 Jan;

[3] Pries LK, Snijders C, Menne-Lothmann C, Decoster J, Winkel RV, Collip D, et al. TwinssCan - Gene-Environment Interaction in Psychotic and Depressive Intermediate Phenotypes: Risk and Protective Factors in a General Population Twin Sample. Twin Research and Human Genetics. 2019 Dec;22(6):460-6.

[4] Myin-Germeys I, Birchwood M, Kwapil T. From Environment to Therapy in Psychosis: A Real-World Momentary Assessment Approach. Schizophrenia Bulletin. 2011 Mar;

[5] Derogatis LR. SCL-90-R: Symptom Checklist-90-R: administration, scoring, and procedures manual. NCS Pearson

[6] Kossakowski J, Groot P, Haslbeck J, Borsboom D, Wichers M. Data from 'Critical Slowing Down as a Personalized Early Warning Signal for Depression'. Journal of Open Psychology Data. 2017 Feb;5(1):1.

[7] Groot PC. Patients can diagnose too: How continuous self-assessment aids diagnosis of, and recovery from, depression. Journal of Mental Health. 2010;19(4):352-62.

[8] Wichers M, Groot PC, Psychosystems, Group ESM, Group EWS. Critical Slowing Down as a Personalized Early Warning Signal for Depression. Psychother Psychosom. 2016;
