# Supplementary material for: Characterizing the dynamics, reactivity and controllability of moods in depression with a Kalman filter
Source: PLoS Comput Biol. 2024 Sep 23;20(9):e1012457. doi: 10.1371/journal.pcbi.1012457 (PMC11449358; doi:10.1371/journal.pcbi.1012457)
Supplement: Attachment — Submitted filename: malamud_reviewer_letter.pdf [file pcbi.1012457.s002.pdf]

Reviewer #1 comments

This paper describes the application of a Kalman filter to model individual differences in self-reported mood dynamics. This is an interesting and clearly written paper showing that a new analytic approach can provide novel insights and incorporate additional information into dynamical systems analyses of mood.

Comments/concerns:

1. The authors briefly cover the use of summary statistics and VAR models to analyze mood dynamics, but does not review any previous work on state space models applied to this sort of data. Have previous studies used Kalman filter or similar state space approaches to study mood dynamics?

   Thank you for your question. In many ways, this paper is the result of our realisation that state space models are rarely if every applied to this sort of data. The most prominent publication in this area is the one by Lodewyckx et al., which we cite. This is particularly true with regards to incorporating external inputs. We have expanded the introduction with some more reference to related literature now (p.5):

   State space models offer a robust, well-established framework that can handle unequal sampling intervals, missing data, and can account for measurement error and inputs to identify rich dynamical phenomena [1, 2, 3]. These models have been applied across various fields, including ecology [4] and neuroscience [5, 6], and can be functionally equivalent to structural equation models, which are widely utilized in psychology [7, 8, 9, 10]. However, despite the increasing theoretical popularity of the dynamic systems perspective [3, 11, 12], state-space models have not been frequently employed to examine mood time series, especially when incorporating external inputs.

2. One motivation the authors provide in the introduction for using model-based analyses is that summary statistics approaches "do not explain the underlying data generation process". Do the authors claim that their model represents the true underlying process generating emotion dynamics?

   Thank you for this question, which hides substantial richness and complexity in its brevity.

   We do clearly state our main motivations for considering the Kalman filter in the penultimate paragraph of the introduction, focusing on unequal time intervals, noise, missing data and inputs. All of these are natural consequence of the Kalman filter being a generative model.

   However, generative emphatically does not mean biologically realistic. Indeed, there are limitations to all parts of the Kalman filter model, and we discuss them in some detail in the limitations section of the discussion. We have now added the following to this limitations section (p.19):

   Finally, we emphasize that while the Kalman filter is a generative model, it remains a descriptive model. It is limited, as noted above, in its expressivity, and its simplistic parameters hide complex underlying cognitive and neurobiological processes which will require richer data and models to characterise and understand.

   In addition, we have clarified the statement in the introduction (p.4):

   Although these statistics are simple and easy to understand, they do not provide a broader framework for understanding and studying mood as they do not capture key aspects of the underlying data generation process.

3. Why did the authors choose minutes as the time steps (versus hours, 15 minute intervals, etc.), given that this leads to very sparse observations? What effects does this choice have on the data, and is there information about the time course of emotion dynamics that can inform the optimal time step?

We defined time steps in the latent state space in minutes, based on the greatest common divisor between the randomly stratified sampling timings of the empirical data. Different time step intervals can indeed alter the underlying dynamics captured by our models. While a 15-minute interval might not drastically affect results, larger intervals could significantly impact the accuracy of our predictions and the estimation of state variables. To examine how the sparsity affects our results, we fitted the Kalman filter to data of 100 participants, which was smoothed over 15-minute, 30-minute, and 60-minute time intervals. The greater the divergence in time gaps, the more the estimates differ (cf Table 1). This makes sense because larger time gaps lead to averaging more data measurement points, resulting in alteration of the data. However, the slowest direction (first eigenvector) remains relatively stable across varying time gaps in the data (cf Table 1 in orange). Additionally, the relationship between the entries of the dynamics matrix and baseline depression scores shows consistency: baseline depression scores were positively correlated with the diagonal elements of the dynamics matrix for negative mood items across all time gaps (cf. Table 2). Our modeling approach not only aligns well with empirical data but also allows for the transformation of estimated parameters into discrete-time parameters for any time interval of interest.

It is challenging to determine the optimal time step in multivariate processes, as the ideal observation interval for one effect depends on all other effects. This interdependence makes it difficult to provide a general recommendation for minimum measurement intervals. Future investigations could explore optimal time step selection based on the specific characteristics of the dynamics under study. Simulation studies could investigate how different time steps affect the accuracy of models in capturing underlying processes.

	ΔT = 1min	ΔT = 15min	ΔT = 30min	ΔT = 60min
ΔT = 1min		0.94 ± 0.07	0.79 ± 0.18	0.38 ± 0.20
ΔT = 15min	0.99 ± 0.005		0.94 ± 0.08	0.62 ± 0.19
ΔT = 30min	0.98 ± 0.02	0.99 ± 0.007		0.80 ± 0.18
ΔT = 60min	0.94 ± 0.06	0.96 ± 0.03	0.97 ± 0.03	

TABLE 1: This table shows in blue the median and the median absolute deviation (MAD) of the correlations between the dynamics matrices estimated on data with different time gaps for 100 participants and in orange the median±MAD of the dot products of the first eigenvector of the dynamics matrices estimated on the data with different time gaps.

	anxious$_t$ $\rightarrow$ anxious$_{t+1}$	sad$_t$ $\rightarrow$ sad$_{t+1}$
ΔT = 1min	$r_{\text{spear}} = 0.23$, $p = 0.02$	$r_{\text{spear}} = 0.32$ $p = 0.001$
ΔT = 15min	$r_{\text{spear}} = 0.24$, $p = 0.01$	$r_{\text{spear}} = 0.23$ $p = 0.02$
ΔT = 30min	$r_{\text{spear}} = 0.21$, $p = 0.03$	$r_{\text{spear}} = 0.29$ $p = 0.004$
ΔT = 60min	$r_{\text{spear}} = 0.22$, $p = 0.03$	$r_{\text{spear}} = 0.28$, $p = 0.006$

TABLE 2: This table shows the Spearman correlations between symptoms and Kalman filter estimates when fitted to data with varying time gaps.

4. The parameter recovery analyses are useful to understand the limitations of the analyses. With more observations, does recovery improve (similar to the first column in figure 2)? Do these analyses provide any recommendations when designing studies to ensure good recovery?

   Recovery of parameters indeed improves with a longer time course of observations. Our simulation study suggested that a minimum of 5-7 days of observations is necessary to achieve a reasonable recovery of the dynamics of interest in a experience sampling setting similar to that of the empirical data. As recovery worsen with including more emotions, we limited our analysis to no more than four emotions. While a longer duration would always be ideal, it also needs to be feasible for participants. Extended study periods may lead to increased missing data due to participant fatigue or disengagement, which presents its own challenges. Incorporating passive data and continuous measures of mood would lead to higher frequency data and thus improve recovery.

   Simulation studies are the best way to inform study design. They allow to test various scenarios and understand the conditions under which parameter recovery is optimized.

5. The controllability analyses are interesting but I had a hard time following the interpretation. How can there be negative controllability (in figure 6B)? I am also having trouble connecting the data in figure 6B to the description in the paragraph below ("how controllable the most controllable direction was appeared to decrease over the three phases (baseline: 4.2, during discontinuation: 1.48, after transition: 1.07))".

   The bar plots in Figure 6B illustrate the singular vectors of the controllability matrix, highlighting the directions in which inputs have the most significant impact on the system. Singular vectors, similar to eigenvectors, can have both positive and negative components, indicating the direction and magnitude of the effect in each coordinate. The scale of an eigenvector is not important, meaning that their signs can be switched. They can point in opposite directions within the vector space. In the context of our study, this means that a driver increasing positive emotions would simultaneously decrease negative emotions, effectively increasing the distinction between positive and negative valence.

   Singular values associated with these vectors indicate the extent to which the system states can be controlled by the inputs. Larger singular values imply that the corresponding directions in the state space (shown in Figure 6B) are more easily controllable. This means that it is possible to influence the system's dynamics along these directions with relatively less input energy. Conversely, lower singular values suggest that more input is needed to influence dynamics along that specific direction.

   This study is based on a single subject, hence the results need to be interpreted with caution. Our primary aim was to demonstrate the power of the control theory approach.

   We hope this explanation clarifies how singular vectors and singular values relate to the controllability of the system and addresses any confusion regarding the negative components in Figure 6B. We added the following to the result section (p.16):

   The most controllable directions varied across different phases (Fig. 6B). Singular vectors, similar to eigenvectors, can have both positive and negative components, indicating the direction and magnitude of the effect in each coordinate, with the ability to switch signs. At baseline, all mood items were aligned in the same direction, making it more difficult to control negative and positive items separately. During the discontinuation phase, the most controllable direction shifted towards more positive items, while negative items became almost uncontrollable. After transitioning to a worse state, the most controllable direction pointed towards a more negative direction, particularly with respect to the "sad" item, and less in a positive direction. Additionally, singular values indicate how easily the system states can be controlled by inputs, with larger values implying easier control and less input energy required to influence the system's dynamics along those directions. We observed that how controllable the most controllable direction was decreased over the three phases (baseline: 4.2, during discontinuation: 1.48, after transition: 1.07), which was expected since more stable systems are less controllable.

Reviewer #2 comments

Thank you for the opportunity to review this manuscript by Malamud and colleagues, which applies a venerable state-space modelling framework (the Kalman filter) to the question of affect dynamics in depression. Three extant datasets were re-analysed using this method, and the results serve as a very compelling proof-of-concept for the application of this analytic framework to this type of data. I was very impressed by the manuscript in general, and have only several relatively minor comments below requesting further elaboration of several aspects of the results.

1. I agree with the manuscript that an important potential bonus of analysing affect dynamics within the state-space modelling framework is the ability of these models to account for the effects of external inputs on subjective affect. Given that potential advantage, therefore, I found it striking that the manuscript found that adding the estimation of input weights to the KF negatively affected inference overall. This is an important point, and should be discussed in more detail in the results (and potentially in the Discussion). In particular, I think more space should be given to discussing exactly which results point to this conclusion (and how), as well as a discussion of the circumstances under which this conclusion would be expected to hold. For instance, is it invariably the case that estimating input weights will negatively affect inference? What about if the subject is placed in an experimental environment where the inputs are exactly controlled and known (e.g., the in-lab modelling paradigm popularised by Robb Rutledge over the last decade or so). No need to do any analyses or simulations to answer these questions, but on reading through I felt it would be important for others to know whether the adverse effects of modelling input are a general feature of the Kalman filter or something more specific to the datasets that were analysed.

    Thank you for emphasising this critical point. We agree that including more data, including inputs, theoretically improves inference. However, in our dataset, the high-dimensional input matrix introduced several challenges. Inputs are complex constructs for a number of reasons. First, it is not clear which latent variable they affect. This means that for every input a 'weight' for every latent variable needs to be estimated. Second, the 'strength' of the input is also challenging to measure, particularly separate from the latent variable and the main dependent variable (the emotional report). This means that the value of the input weight itself also needs to be estimated. Third, the precise timing of the input and its duration is also important but unknown or at least hard to measure well. Finally, inputs are also necessarily sparsely measured. Overall this leads to a situation where reliable estimation will likely always be challenging.

    There are several possible approaches. First, one can consider a stronger criterion for excluding inputs with low frequency might be advisable. For example, excluding inputs that occur below a certain threshold per subject could help, although this approach risks excluding rare but potentially significant inputs.

    Another interesting direction is a hierarchical extension of the Kalman filter, which would allow some shared information between individuals, and we refer to this in the limitation section (p.18):

    In order to improve recoverability in higher-dimensional systems, parameters need to be constrained using e.g. lasso-restrictions for the input weights. Another potential approach consists in using multi-level (hierarchical) models. However, [13] also reported overestimated error variances and lower dynamic parameters using a hierarchical state-space model.

    In line with your suggestion, we have thought about experimental environments where inputs are controlled and known, similar to Robb Rutledge's emotion triggering studies. To explore this, we conducted a separate study [14] where participants reported their multidimensional emotional state while watching short emotional video clips. This controlled setting, with equal sampling intervals and dense mood and input data, indeed

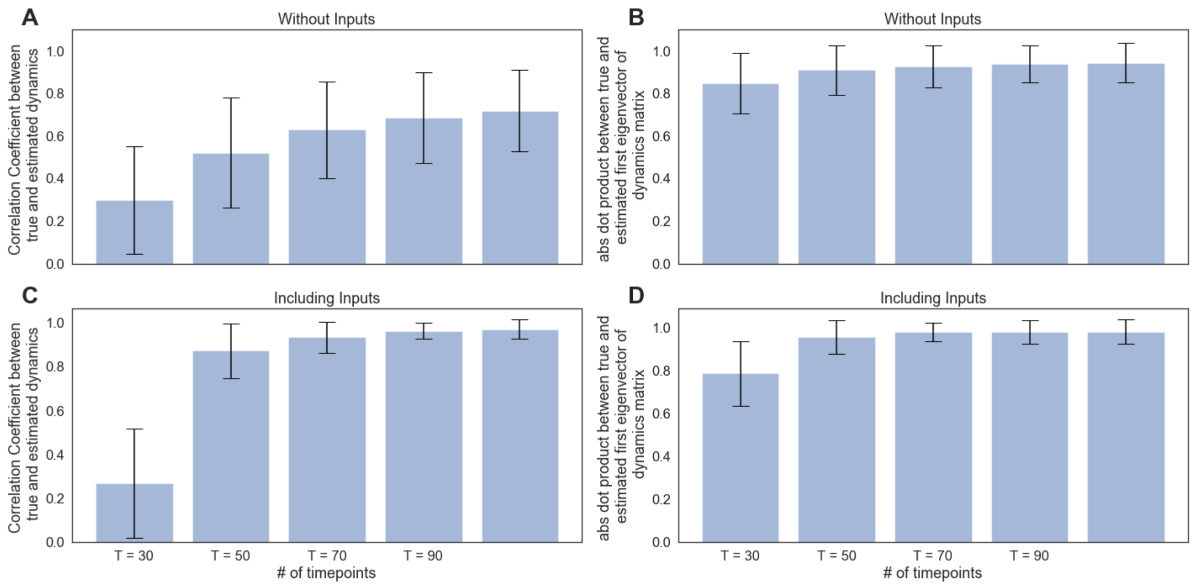

FIGURE 1: Illustration of the recoverability of the dynamics matrix, with **A)** showing the correlation between entries of the known and estimated dynamics matrix, and **B)** showing the absolute dot product of the known and estimated first eigenvector of the dynamics matrix when simulating data without inputs. **C)** & **D)** show the same metrics for simulated data with inputs. The results demonstrate that incorporating inputs enhances the recoverability of the dynamics matrix.

showed better performance when including inputs, as seen in the added figure from that study's simulation data (cf Fig 1).

In conclusion, the inclusion of inputs does not necessarily worsen the performance of the Kalman filter, but empirically it often might. The observed adverse effects in our study are due to the quality and structure of the data. This underscores the importance of understanding and improving these measurement aspects to enable a more comprehensive and accurate assessment of mood and psychological constructs in general.
We have added the following to the results section (p.9&11):
Adding estimation of input weights **C** affected inference advsersely. This is because it added a large number of parameters to the estimation, and these are difficult to estimate due to the high dimensionality and extreme sparsity of the input matrix. However, it is important to remember that the apparently better performance on the data without inputs is false to the extent that it does not capture the misestimation due to the missing inputs.

Additionally, we have expanded the discussion section to include (p.17&18): In two of the datasets analysed here, information about participants' context at the time of the measurement was provided, which allowed us to expand our model to a control system. The Kalman filter performance worsened when including this contextual information as external inputs, which may seem counterintuitive as more data (inputs) should improve the model's accuracy. However, the inclusion of data also requires extending the model and adding a large number of parameters. The ratio of additional parameters to additional data is not, unfortunately, conducive to more precise inference. We note, though that the apparently better performance without inputs is misleading because it incorrectly ascribes change driven by external inputs to internal dynamics. This highlights the

importance and challenge of effectively measuring emotions and the immediate context.

2. Very little of the manuscript's discussion is concerned with interpreting the clinical significance of results for theories of depression. I appreciate that this is not the primary focus of the manuscript, but even so I feel that the authors are well placed here to draw some links between their findings and the extant literature on affect dynamics in depression (e.g., work on inertia of affect in depression). I think it would be helpful for readers to have access to an expanded discussion of these effects in the context of psychiatric theory.

   Thank you for your suggestions. We added the following to the discussion (p.18):
   To conclude, how do our findings relate to theories of psychopathology? Depression is characterized by a predominance of negatively valenced, low-arousal emotions such as sadness and lethargy, with a well-documented persistence of these negative mood states (high emotional inertia) [15, 16]. In our study, we found that individuals with depression not only experience more stable patterns of negative mood but also exhibit less emotional flexibility. This observation sits well with cognitive theories of depression, which propose that ruminative thought patterns contribute to this affective inertia, making it difficult for individuals to transition out of negative emotions [17]. Negative affect can persist even in the presence of potentially mood-enhancing events. This is understandable because we observed that in individuals with higher levels of depression the direction requiring the least input effort tends to shift more towards negative moods and less towards positive moods. Therefore, it would require significant effort to achieve a positive mood shift. Behaviorally, reduced engagement in rewarding activities – a core aspect of behavioral activation theory – further exacerbates this issue, as individuals with depression are less likely to seek out or participate in activities that could enhance their mood[16].

   We hope that provides a clearer linkage between our findings and the broader literature on affect dynamics in depression.

3. Typo on page 7, line 171: the manuscript uses the word "punctuate" rather than "punctate", which I think is what is intended

4. The labelling of subplots in Figure 1 is rather confusing; for instance, subplot B itself includes two subplots themselves labelled a and b. This has the potential to confuse readers and a more unambiguous labelling system should be chosen.

   Thanks for pointing this out. Both issues were addressed in the manuscript.

References

[1] Staudenmayer J, Buonaccorsi JP. Measurement Error in Linear Autoregressive Models. Journal of the American Statistical Association. 2005 Sep;100(471):841-52. Publisher: Taylor & Francis _eprint: https://doi.org/10.1198/016214504000001871. Available from: https://doi.org/10.1198/016214504000001871.

[2] Durbin J, Koopman SJ. Time Series Analysis by State Space Methods: Second Edition. Oxford Statistical Science Series. OUP Oxford; 2012. Available from: https://books.google.ch/books?id=fOq39Zh0olQC.

[3] Durstewitz D, Huys QJM, Koppe G. Psychiatric Illnesses as Disorders of Network Dynamics. Biological Psychiatry: Cognitive Neuroscience and Neuroimaging. 2020. Publisher: Elsevier BV.

[4] Newman K, King R, Elvira V, de Valpine P, McCrea R, Morgan B. State-space Models for Ecological Time Series Data: Practical Model-fitting. Methods in Ecology and Evolution. 2022 Mar;14.

[5] Durstewitz D, Koppe G, Thurm MI. Reconstructing computational system dynamics from neural data with recurrent neural networks. Nature Reviews Neuroscience. 2023 Nov;24(11):693-710.

[6] Koppe G, Guloksuz S, Reininghaus U, Durstewitz D. Recurrent Neural Networks in Mobile Sampling and Intervention. Schizophrenia Bulletin. 2019;45:272-6.

[7] Chow SM, Ho MH, Hamaker E, Dolan C. Equivalence and Differences Between Structural Equation Modeling and State-Space Modeling Techniques. Struct Equ Model. 2010 Apr;17.

[8] Epskamp S, Rhemtulla M, Borsboom D. Generalized Network Psychometrics: Combining Network and Latent Variable Models. Psychometrika. 2017 Dec;82(4):904-27. Available from: https://doi.org/10.1007/s11336-017-9557-x.

[9] Kushner MG, Krueger RF, Wall MM, Maurer EW, Menk JS, Menary KR. Modeling and treating internalizing psychopathology in a clinical trial: a latent variable structural equation modeling approach. Psychological medicine. 2013 Aug;43:1611-23.

[10] Kendler KS, Kessler RC, Neale MC, Heath AC, Eaves LJ. The prediction of major depression in women: toward an integrated etiologic model. Am J Psychiatry. 1993 Aug;150(8):1139-48.

[11] Scheffer M, Bockting CL, Borsboom D, Cools R, Delecroix C, Hartmann JA, et al. A Dynamical Systems View of Psychiatric Disorders-Practical Implications: A Review. JAMA psychiatry. 2024 Jun;81(6):624-30.

[12] Bringmann L, Helmich M, Eronen M, Voelkle M. Complex Systems Approaches to Psychopathology. In: Krueger RF, Blaney PH, editors. Oxford Textbook of Psychopathology. Oxford Textbook of Psychopathology. Oxford University Press; 2023. p. 103-22.

[13] Lodewyckx T, Tuerlinckx F, Kuppens P, Allen NB, Sheeber L. A hierarchical state space approach to affective dynamics. Journal of Mathematical Psychology. 2011;55(1):68-83. Publisher: Elsevier BV.

[14] Malamud J, Huys Q. Distancing alters the controllability of emotional states by altering both intrinsic stability and extrinsic sensitivit; 2023. Type: article. Available from: https://doi.org/10.21203/rs.3.rs-2731985/v1.

[15] Brose A, Schmiedek F, Koval P, Kuppens P. Emotional inertia contributes to depressive symptoms beyond perseverative thinking. Cognition & Emotion. 2015;29(3):527-38.

[16] Kuppens P, Allen NB, Sheeber LB. Emotional inertia and psychological maladjustment. Psychol Sci. 2010 Jul;21(7):984-91. Available from: http://dx.doi.org/10.1177/0956797610372634.

[17] Houben M, Noortgate WVD, Kuppens P. The relation between short-term emotion dynamics and psychological well-being: A meta-analysis. Psychological Bulletin. 2015 Jul;141(4):901-30. Publisher: American Psychological Association Inc.
